# Dose‐effect relationship between brisk walking and blood pressure in Chinese occupational population with sedentary lifestyles: Exercise and blood pressure

**DOI:** 10.1111/jch.14340

**Published:** 2021-08-13

**Authors:** Yingxiang YU, Cuiqing CHANG, Yifan WU, Chengcheng GUO, Lan XIE

**Affiliations:** ^1^ Institute of Sports Medicine Peking University Third Hospital Beijing China

**Keywords:** blood pressure, brisk walking, sedentary lifestyle

## Abstract

The purpose of this study was to explore the effects of brisk walking on blood pressure (BP) in Chinese Han occupational populations to identify the appropriate volume of exercise needed for BP management. Eight‐hundred and two office staff with sedentary lifestyles were recruited to receive a prescription pedometer‐assisted brisk walking intervention for 3 months. To evaluate exercise intervention efficiency using statistical methods, the effective steps was divided as follows: 4000‐ < 8000 (Level 1), 8000‐ < 10 000 (Level 2), 10 000–12 000 (Level 3), and > 12 000 (Level 4) steps/day. The data of 688 participants who completed the study were analyzed. After intervention, Systolic BP (SBP) and Diastolic BP (DBP) at Levels 1–3 were significantly decreased (*p* < .05). For participants with hypertension at baseline, all four levels demonstrated a significantly reduced SBP and DBP. In addition, there was a significant dose‐effect relationship between the effective steps and the SBP. Compared with the maximum effective steps level (Level 4), the average change of SBP between level 1–3 and level 4 were statistically significant, with ‐3.24 mm Hg (95%CI: ‐5.74 to ‐0.74, *p* = .011), ‐2.58 mm Hg (95%CI: ‐4.73 to ‐0.43, *p* = .019), and ‐2.19 mm Hg (95%CI: ‐4.20 to ‐0.18, *p* = .033), respectively. For the hypertensive cohort, only the difference between Level 1 and 4 was statistically significant (Level 1 vs. Level 4: difference in means = ‐6.22 mm Hg, 95%CI: ‐12.68 to ‐0.24, *p* = .036). No significant dose‐effect relationship of DBP was observed. Our findings showed brisk walking can effectively control BP in Chinese populations and a significant dose‐effect relationship was found between exercise and SBP.

## 1 INTRODUCTION

The prevalence of hypertension is increasing yearly, with worldwide cases estimated to increase by 15–20%, to 1.5 billion by 2025.^1^ According to the Chinese Hypertension Survey, from 2012 to 2015, approximately 244.5 million Chinese people aged ≥18 years suffered from hypertension.^2^ Hypertension, especially elevated systolic blood pressure (SBP), is a key risk factor for morbidity and mortality worldwide, with approximately 9 million deaths attributed to the condition each year.^3^, ^4^, ^5^ Although cardiovascular and cerebrovascular diseases, hypertensive cardiovascular diseases, have become a major economic burden worldwide,^3^, ^6^ their prevalence may be reduced through efficient control of hypertension, their most preventable risk factor.^7^

Multiple studies have shown that increasing physical activity can significantly reduce SBP and diastolic blood pressure (DBP),^8^, ^9^ thereby significantly reducing the incidence of both cardiovascular and cerebrovascular events in patients with hypertension; thus, improving quality of life and reducing the burden of disease.^4^, ^10^, ^11^ The benefits of exercise on health are well established, with physical activity guidelines in different countries recommending various exercises for different health targets.^3^, ^12^, ^13^ Healthy individuals seeking to maintain overall fitness are generally advised to participate in at least 30 min of moderate‐intensity exercise daily, accumulating at least 150 min of weekly exercise. Further, to achieve superior health benefits, or to reduce cardiovascular disease risk, it is recommended that individuals participate in 300 min of moderate or 150 min of vigorous exercise per week. European Society of Cardiology/European Society of Hypertension guidelines^1^ recommend that patients with hypertension engage in at least 30 min of moderate‐intensity aerobic exercise 5–7 days per week; equivalent to brisk walking for at least 3000 step counts per day (a minimum of 100 steps/min for at least 30 min).^14^ However, these guidelines do not explicitly indicate the upper limit of exercise required to improve health outcomes, or the effects of exercise duration on health benefits, especially in relation to cardiovascular health.

Although the positive effects of increased exercise on blood pressure (BP) and other cardiovascular disease risk factors are well established, there is limited research investigating the optimal amount of exercise required to prevent or control hypertension across various ethnic groups, sexes, ages, and occupations; thus, necessitating further study. Herein, we investigate the optimal amount of exercise required to prevent or control hypertension in the Chinese Han office worker population, thereby providing a scientific basis for the application of exercise interventions in BP management in the Chinese population.

## 2 METHODS

### 2.1 Participants

A total of 802 participants, comprising of 398 males and 404 females, were recruited from various enterprises and institutions. Inclusion criteria were as follows: (1) full‐time employees; (2) aged 18–65 years; and (3) a sedentary lifestyle involving six or more hours of sitting per day. Exclusion criteria were as follows: (1) poor BP control (systolic > 180 mm Hg and/or diastolic > 110 mm Hg); (2) a history of diagnosed bone and joint diseases; (3) a history of spinal or limb surgery, or fractures within the last 3 months; (4) serious dysfunction, or organic diseases, of the heart, liver, or kidneys; (5) a known HIV history, active hepatitis B/C infection or an uncontrolled active systemic infection; and (6) pregnant or lactating women.

This study was approved by the Peking University Third Hospital Medical Science Research Ethics Committee (No. [2014]189 (2)) and registered at the China Clinical Trial Registration Center (Registration no. ChiCTR‐OOh‐16010229). All participants were informed of the purpose, content, and risks involved with study participation; written informed consent was collected prior to trial commencement.

### 2.2 Study design

This study included all participants in the brisk walking intervention, and those with under or beyond physical activity recommendations were considered as the reference group (control group). Furthermore, this is a self‐controlled study, in which participants acted as their own controls when pre‐intervention and post‐intervention BP were measured. Prior to intervention, all participants were educated in the dietary management of BP via a single information session. Next, each participant was prescribed a 3‐month pedometer‐assisted, self‐monitored walking intervention.

### 2.3 Brisk walking intervention program

Exercise intervention program is based on open‐ended walking interventions in a real world. Brisk walking was the chosen form of exercise in this study. An exercise suggestion is proposed to participants based on their original physical activity levels (PALs), and the established internet‐based exercise and nutrition health management application and a prescription pedometer were used to implement the exercise intervention, as well as to guide and record all involvements in exercise.^15^

The intervention program included an exercise intensity (moderate walking speed of 80–90 meters/min for females (4.0‐4.5 MET) and 90–100 meters/min for males (4.5‐5.0 MET)^16^) equivalent of a pedometer reading of 100–150 steps/min,^14^ an exercise duration of at least 10 min/bout, daily cumulative exercise time of 30–80 min, an exercise frequency of 5–7 days/week, an exercise execution time period between 6:00 and 23:00, optimal time of exercise, for example, avoiding morning exercise in hypertensive patients, and precautions during exercise. For the first month, walking programs were set according to a participant's past PALs, which, if found low (eg, no or very little exercise), required that participants be careful and exercise as much as possible in the first 2 weeks of initiating exercise. Participants were to gradually increase exercise time and exercise intensity to ensure safety during the exercise and to prevent sports‐related injuries; eventually all participants were encouraged to exercise whenever possible. Although this 30‐min could be completed in one or several sessions, the duration of each continuous exercise was to be a minimum of 10 min. In the second or third months, exercise targets were gradually increased according to the previous month's completion. In principle, the exercise intervention program in this study met the recommended amount of exercise set by the guidelines for the management of hypertension.^1^

### 2.4 Measurements and procedures

All participants underwent outcome measurements at baseline and study completion.

#### 2.4.1 Blood Pressure

BP was measured in the right arm using a regularly calibrated mercury sphygmomanometer, with accordance to the 2010 Chinese Guidelines for the Management of Hypertension.^17^ Each participant's BP was measured in triplicate, with 60‐s intervals between each measurement, and the average used as the final value. BP was measured in mm Hg.

#### 2.4.2 General physical examination indicators

Height, body weight (BW), and waist circumference (WC) were measured using standard methods. Body mass index (BMI, kg/m^2^) = body weight (kg)/height (m) ^2^. Body fat mass was measured using a MC‐180 body composition analyzer (Tanita Corp., Japan). Body fat percentage (BF %) = body fat mass (kg)/body weight (kg)×100%. The body composition analyzer was calibrated before each use.

#### 2.4.3 Physical Activity Level

Prior to intervention implementation, information regarding each participants' exercise habits was collected and their individual exercise risks established. According to their exercise habits in the past 6 months, each participant was graded into one of the following four PAL levels: PAL1: No or very little exercise; PAL2: Moderate or above intensity exercise, accumulating > 30 min/day, less than 3 days/week; PAL3: Moderate or above intensity exercise, accumulating > 30 min/day, at least 3 days/week, regular exercise for < 6 months; PAL4: Moderate or above intensity exercise, accumulating > 30 min/day, at least 3 days/week, regular exercise for 6 months or more.

#### 2.4.4 Walking evaluation

Step counts accumulated with a frequency of 100–150 steps/min were considered as "effective steps” and recorded using a prescription pedometer.^15^ Participants were, therefore, required to wear a pedometer daily throughout the trial period. Additionally, each participant's walking program was recommended for 30–80 min/day; thus, the recommended number of effective steps was 3000–12 000 steps/day. Thus, for statistical analysis, we divided the effective steps achieved by each participant into the following four levels: 4000‐ < 8000 (Level 1), 8000‐ < 10 000 (Level 2), 10 000–12 000 (Level 3), and > 12 000 (Level 4) steps/day. As Level 4 was beyond physical activity recommendations, it was established as the reference group against which the efficacy of the intervention, and thus the recommended amount of exercise, was evaluated and verified.

#### 2.4.5 Diet scores

The eating behavior section of the Healthstyle: A Self‐Test^18^ (derived from the United States Public Health Service) was used to evaluate the dietary choices of participants. This questionnaire assessed eating rationale based on the diversity of a participant's daily diet, whether it consciously restricted fat intake (especially saturated and trans fatty acids), salt, and sugar. The score range was 0–10.

#### 2.4.6 Diagnostic criteria of hypertension

Hypertension was defined as SBP ≥140 mm Hg and/or DBP ≥90 mm Hg, or an existing history of hypertension and current administration of antihypertensive therapy.^17^

### 2.5 Statistical analysis

IBM SPSS version 23.0 (IBM Corp., Armonk, NY, USA) and R version 3.6.1 (R Core Team. R: A language and environment for statistical computing. R Foundation for Statistical Computing, Vienna, Austria. URL https: //www.R‐project.org/, 2019) were used for statistical analysis. *p* < .05 was deemed statistically significant.

Normally distributed (Kolmogorov–Smirnov test) data were expressed as mean ± standard deviation (M±SD). An independent sample *t*‐test was used for comparison between two groups, while a paired sample *t*‐test was used to compare outcomes before and after intervention in a single group. Multiple linear regression models were used to analyze the main influencing factors of BP change (BP of post intervention minus BP of pre intervention), including sex, age, height, baseline PAL, changes in BW, WC, BF%, diet scores, and antihypertensive medication use. *p* < .05 was considered statistically significant.

## 3 RESULTS

### 3.1 Participant characteristics and exercise performance

**FIGURE 1:**
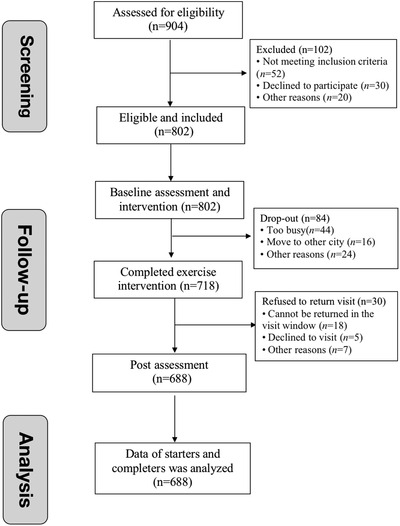
The study flow diagram

Of the 802 patients recruited to this study, 114 withdrew due to personal reasons (Figure 1); data of the remaining 688 participants, 331 males and 357 females, were analyzed. The average age was 37.0±9.9 years, with no significant differences between sexes (*p* = .54). General participant characteristics at baseline are shown in Table 1, with significant differences existing between the height, BW, BMI, WC, and BF% (*p* < .05) of males and females. 17.0% (117/688) of participants had a diagnosis of hypertension; 49 of which were undergoing antihypertensive therapy.

**TABLE 1:** General Baseline characteristics of patients

	Total group	Males	Females	*p* value^a^
N	688	331	357	
Age (year)	37.0±9.9	36.7±9.6	37.2±10.1	.540
Height (cm)	166.2±8.0	172.5±5.5	160.4±5.0	<.001
BW (kg)	66.5±13.9	74.9±12.4	58.7±10.2	<.001
BMI (kg/m^2^)	23.9±3.9	25.1±3.7	22.8±3.8	<.001
WC (cm)	82.9±11.8	88.8±10.6	77.4±10.1	<.001
BF% (%)	27.1±6.8	24.2±5.4	29.6±6.8	<.001
Diet scores	5.3±2.6	5.1±2.5	5.4±2.7	.022
Hypertensive (*n*, %)	anti‐hypertensive therapy	49 (7.1)	32 (9.7)	17 (4.8)
	None	68 (9.9)	50 (15.1)	18 (5.0)

Data are expressed as mean ± SD.

*Abbreviations: N*, number of participants; BW, Body weight; BMI, body mass index; WC, waist circumference; BF%, body fat percentage.

^a^ Comparisons between males and females.

**TABLE 2:** Changes of blood pressure and body weight in different effective steps levels

			^a^Effective step levels			
			Level 1	Level 2	Level 3	Level 4
Total	*N*		92	174	237	185
	SBP (mm Hg)	Pre	121.4±15.0	118.1±14.7	115.4±13.4	116.4±13.3
		Post	116.9±11.2	114.7±13.2	113.4±11.9	117.3±12.2
		Δ (post‐pre)	−4.5±12.0	−3.4±9.8	−2.0±10.5	0.9±10.5
		*p*‐value	<.001	<.001	.005	.241
	DBP (mm Hg)	Pre	79.3±12.8	77.2±11.6	75.1±10.5	76.1±10.1
		Post	75.9±9.1	74.9±10.7	73.4±9.6	74.9±9.7
		Δ (post‐pre)	−3.4±9.7	−2.3±8.9	−1.7±8.6	−1.2±9.2
		*p*‐value	.001	.001	.003	.078
	BW (kg)	Pre	71.9±16.7	64.6±13.2	65.3±14.4	67.1±11.6
		Post	70.2±16.0	63.7±12.6	64.4±13.5	66.3±11.5
		Δ (post‐pre)	−1.3±2.9	−0.9±2.9	−0.9±3.2	−0.8±2.7
		*p*‐value	<.001	<.001	<.001	<.001
Male	*N*		51	71	102	107
	SBP (mm Hg)	Pre	123.4±13.5	126.8±11.4	121.2±12.2	120.7±12.4
		Post	120.3±9.7	123.2±11.2	119.5±10.3	120.9±11.1
		Δ (post‐pre)	−3.1±12.9	−3.6±9.0	−1.7±10.5	0.2±9.5
		*p*‐value	.090	.001	.099	.793
	DBP (mm Hg)	Pre	81.7±13.4	83.3±10.5	78.5±10.0	79.5±9.6
		Post	79.0±8.8	81.0±8.7	76.1±8.8	77.4±9.5
		Δ (post‐pre)	−2.7±10.6	−2.3±9.5	−2.4±8.0	−2.1±9.0
		*p*‐value	.068	.041	.004	.019
	BW (kg)	Pre	77.5±17.2	73.5±12.0	76.0±12.6	73.6±9.2
		Post	75.9±16.1	72.4±11.5	74.4±11.4	72.5±9.0
		Δ (post‐pre)	−1.6±3.5	−1.1±2.6	−1.6±4.1	−1.0±2.8
		*p*‐value	.002	.001	<.001	<.001
Female	*N*		41	103	135	78
	SBP (mm Hg)	Pre	118.8±16.4	112.2±13.7	110.9±12.6	110.5±12.3
		Post	112.5±11.5	108.8±11.1	108.8±11.0	112.3±12.0
		Δ (post‐pre)	−6.3±10.7	−3.4±10.3	−2.1±10.6	1.8±11.7
		*p*‐value	.001	.001	.023	.173
	DBP (mm Hg)	Pre	76.3±11.4	73.1±10.5	72.6±10.1	71.4±8.9
		Post	72.0±8.0	70.7±9.9	71.4±9.7	71.4±9.0
		Δ (post‐pre)	−4.3±8.6	−2.4±8.5	−1.2±9.0	0.0±9.4
		*p*‐value	.003	.005	.123	1.000
	BW (kg)	Pre	65.0±13.2	58.4±10.1	57.3±9.7	58.2±8.2
		Post	64.1±13.3	57.6±9.5	56.9±9.5	57.7±8.5
		Δ (post‐pre)	−0.9±1.9	−0.8±3.1	−0.4±2.1	−0.4±2.6
		*p*‐value	.005	.011	.031	.136

Data are expressed as mean ± SD.

^a^ Effective steps levels, Level 1: 4000 ‐ < 8000 Steps/day; Level 2: 8000 ‐ < 10 000 Steps/day; Level 3: 10 000–12 000 Steps/day; Level 4: more than12 000 Steps/day.

*Abbreviations*: BW, body weight; DBP, diastolic blood pressure; *N*, number of participants; SBP, systolic blood pressure; Δ, is a 3‐month change.

The effective steps for all 688 participants who completed the exercise intervention was as follows: percentage of participants with 4000‐ < 8000, 8000‐ < 10 000, 10 000–12 000, and > 12 000 effective steps/day was 13.4%, 25.3%, 34.4%, and 26.9%, respectively.

### 3.2 Effects of brisk walking on BP and its dose‐effect relationship

For all participants, as shown in Table 2, the effective steps at Level 1–3, both of SBP and DBP were significantly reduced after 3 months (*p* < .05); changes of SBP and DBP at Level 4 were not significant, especially the average SBP, instead showed a rising trend. For the male cohort, SBP decreased significantly by 3.1 mm Hg (*p* = .010) and 3.6 mm Hg (*p* = .001) on average at Level 1 and 2, respectively; and the SBP changes at the other effective step levels were not significant. DBP showed a decrease at Level 1–4, but it is only statistically significant in Level 2–4 (*p* < .05). For the female group, SBP decreased significantly in Level 1–3, with an average decrease of 6.3 mm Hg (*p* = .001), 3.4 mm Hg (*p* = .001), and 2.1 mm Hg (*p* = .023) respectively; however, SBP at Level 4 increased with an average of 1.8 mm Hg (*p* = .173). DBP showed a significant decrease in Level 1 and Level 2, by 4.3 mm Hg (*p* = .003) and 2.4 mm Hg (*p* = .005), respectively. However, Level 3 and 4 did not change significantly. In addition, there was a significant reduction in body weight at all four effective steps levels.

The BP change was taken as the dependent variable, and the possible related factors of BP changes were taken as independent variables for multivariate analysis, as shown in Table 3. In the analysis, the entry regression method was used to fit the model, and the test level was 0.05. There was a dose‐effect relationship between the change of SBP and the effective steps (Table 4). Compared with the highest category of effective steps (Level 4), the difference of SBP change was ‐3.24 mm Hg (95% CI: ‐5.74 to ‐0.74, *p* = .011) for the lowest (Level 1) category, ‐2.58 mm Hg (95% CI: ‐4.73 to ‐0.43, *p* = .019) for Level 2, and ‐2.19 mm Hg (95% CI: ‐4.20 to ‐0.18, *p* = .033) for level 3. For the male cohort, the difference of SBP changes between Level 1 and Level 4 was significant (‐3.95 mm Hg, 95% CI: ‐7.35 to 0.55, *p* = .024). For female, it only Level 2 showed a significant difference (‐3.41 mm Hg, 95% CI: ‐6.48 to ‐0.34, *p* = .030). However, there was no significant dose‐effect relationship between the effective steps and the changes of DBP.

**TABLE 3:** Multiple linear regression analysis of blood pressure changes (n = 688)

	Variable	Coefficients β value	Std. error	*t*‐value	*p*‐value
**SBP change** ^a^	Sex (male = 1,female = 0)	2.024	1.496	1.352	.177
	Age	−0.107	0.051	−2.100	.036
	Height	−0.131	0.090	−1.456	.146
	Baseline PAL	0.784	0.523	1.499	.135
	Hypertensive(yes = 1, no = 0)	−4.905	1.928	−2.544	.011
	Effective steps	0.001	0.000	3.750	.000
	Δ BW	0.618	0.188	3.295	.001
	Δ WC	−0.089	0.124	−0.719	.472
	Δ BF%	0.125	0.167	0.747	.455
	Δ eating score	0.101	0.146	0.688	.492
**DBP change** ^b^	Sex (male = 1,female = 0)	−0.327	1.277	−0.256	.798
	Age	−0.114	0.043	−2.635	.009
	Height	−0.039	0.077	−0.506	.613
	Baseline PAL	0.434	0.447	0.972	.332
	Hypertensive(yes = 1, no = 0)	−2.148	1.646	−1.304	.193
	Effective steps	0.000	0.000	2.233	.026
	Δ BW	0.191	0.160	1.189	.235
	Δ WC	−0.075	0.106	−0.703	.482
	Δ BF%	0.119	0.143	0.832	.406
	Δ eating score	0.117	0.125	0.941	.347

^a^ Dependent Variable: SBP change.

^b^ Dependent Variable: DBP change.

*Abbreviations*: Δ, is a 3‐month change (post‐pre); PAL, physical activity level; BW, Body weight; WC, waist circumference; BF%, body fat percentage.

**TABLE 4:** The dose‐effect relationship between changes of blood pressure and effective steps levels

^a^Effective steps levels	ΔSBP^b^	*p*‐value	ΔDBP^c^	*p*‐value
**Total group**				
Level 1 versus 4	−3.24 (‐5.74,‐0.74)	.011	−0.74 (‐2.88,1.41)	.503
Level 2 versus 4	−2.58 (‐4.73,‐0.43)	.019	0.12 (‐1.74,1.98)	.899
Level 3 versus 4	−2.19 (‐4.20,‐0.18)	.033	0.54 (‐1.20,2.28)	.542
**Males**				
Level 1 versus 4	−3.95 (‐7.35,‐0.55)	.024	0.16 (‐2.67,2.98)	.914
Level 2 versus 4	−1.39 (‐4.52,1.74)	.384	1.78 (‐0.82,4.39)	.181
Level 3 versus 4	−2.26 (‐5.16,0.65)	.129	0.12 (‐2.31,2.56)	.922
**Females**				
Level 1 versus 4	−2.68 (‐6.39,1.03)	.158	−2.02 (‐5.25,1.21)	.222
Level 2 versus 4	−3.41 (‐6.48,‐0.34)	.030	−1.33 (‐4.03,1.37)	.334
Level 3 versus 4	−2.25 (‐5.12,0.63)	.126	0.24 (‐2.28,2.76)	.854

Data are expressed as mean difference (95% confidence interval).

^a^ Effective steps levels, Level 1: 4000 ‐ < 8000 Steps/day; Level 2: 8000 ‐ < 10 000 Steps/day; Level 3: 10 000‐ 12 000 Steps/day; Level 4: more than 12 000 Steps/day.

^b^ ΔSBP: difference of systolic blood pressure change in mm Hg (95% confidence interval).

^c^ ΔDBP: difference of diastolic blood pressure change in mm Hg (95% confidence interval).

### 3.3 Effect of different effective steps levels on blood pressure of hypertensive and non‐hypertensive participants

According to the presence of hypertension, participants were divided into the hypertensive and the non‐hypertensive groups for subgroup analysis (Table 5). For the hypertensive cohort, after 3 months of brisk walking, SBP and DBP both significantly decreased at Levels 1 to 4 (*p* < .05). For non‐hypertensive cohort, SBP decreased in levels 1–3; however, only Level 2 (‐2.2±9.1 mm Hg, *p* = .004) was significant. On the other hand, Level 4 had a significant increase (2.0±10.2 mm Hg, *p* = .015). The change of DBP was not significant.

**TABLE 5:** Blood pressure changes of hypertensive and non‐hypertensive participants at different effective step levels

		Hypertensive					Non‐hypertensive				
^a^Effective steps levels		*N*	Pre	Post	Δ (post‐pre)	*p*‐value	*N*	Pre	Post	Δ (post‐pre)	*p*‐value
Level 1	SBP	27	137.0±10.7	125.1±8.4	−11.8±13.4	<.001	65	114.9±11.3	113.4±10.4	−1.5±10.0	.229
	DBP	27	93.8±8.9	82.9±7.6	−10.9±9.7	<.001	65	73.3±8.6	73.0±8.0	0.3±7.9	.744
Level 2	SBP	33	137.1±11.1	128.3±12.1	−8.8±11.0	<.001	141	113.7±11.5	111.5±11.3	−2.2±9.1	.004
	DBP	33	92.9±9.7	84.1±9.7	−8.8±8.4	<.001	141	73.6±8.6	72.7±9.7	−0.9±8.4	.217
Level 3	SBP	31	135.5±13.3	127.4±12.7	−8.1±12.1	.001	206	112.3±10.5	111.3±10.3	−1.0±10.0	.144
	DBP	31	90.9±7.5	83.9±8.9	−7.0±10.1	.001	206	72.8±8.6	71.8±8.7	−0.9±8.1	.107
Level 4	SBP	26	134.3±12.5	128.6±12.2	−5.7±9.7	.006	159	113.4±10.9	115.4±11.2	2.0±10.2	.015
	DBP	26	89.4±9.9	84.3±10.4	−5.1±10.1	.016	159	73.9±8.3	73.4±8.8	−0.5±8.9	.430

Data are expressed as mean ± SD in mm Hg.

^a^ Effective steps levels, Level 1: 4000 ‐ < 8000 Steps/day; Level 2: 8000 ‐ < 10000 Steps/day; Level 3: 10000‐ 12000 Steps/day; Level 4: more than 12000 Steps/day.

*Abbreviations*: N, number of participants; SBP, systolic blood pressure; DBP, diastolic blood pressure; Δ, is a 3‐month change.

The multiple linear regression model was used to analyze the relationship between BP and exercise. The results show that there was a significant dose‐effect relationship between the change in SBP and the effective steps (Table 6). Compared to Level 4, only the difference of Level 1 is statistically significant, which is ‐6.22 mm Hg (95% CI: ‐12.68 to ‐0.24, *p* = .036); the other levels were not significantly different. There was no significant dose‐effect relationship between DBP and the effective steps. For the non‐hypertensive, as shown in Table 5, there was a significant dose‐effect relationship between the change of SBP and the effective steps, but no significant dose‐effect relationship was found in DBP.

**TABLE 6:** Relationship between the change (post‐pre) of blood pressure and effective step levels in hypertensive and non‐hypertensive participants

	Hypertensive				Non‐hypertensive			
^a^Effective steps levels	ΔSBP^b^	*p*‐value	ΔDBP^c^	*p*‐value	ΔSBP^b^	*p*‐value	ΔDBP^c^	*p*‐value
Level 1 versus 4	−6.22 (‐12.68,0.24)	.036	−4.79 (‐9.95,0.36)	.073	−3.04 (‐5.88,‐0.19)	.037	−0.08 (‐2.57,2.41)	.950
Level 2 versus 4	−3.73 (‐9.96,2.51)	.245	−3.51 (‐8.56,1.55)	.179	−2.54 (‐4.88,‐0.20)	.034	0.68 (‐1.37,2.74)	.5167
Level 3 versus 4	−1.16 (‐8.05,5.73)	.742	0.32 (‐5.17,5.8)	.911	−2.32 (‐4.44,‐0.19)	.033	0.58 (‐1.29,2.44)	.547

Data are expressed as mean difference (95% confidence interval).

^a^ Effective steps levels, Level 1: 4000 ‐ < 8000 Steps/day; Level 2: 8000 ‐ < 10 000 Steps/day; Level 3: 10 000‐ 12 000 Steps/day; Level 4: more than 12 000 Steps/day.

^b^ ΔSBP: difference of systolic blood pressure change in mm Hg (95% confidence interval).

^c^ ΔDBP: difference of diastolic blood pressure change in mm Hg (95% confidence interval).

## 4 DISCUSSION

This study showed that 3 months of brisk walking, with a pace of 100–150 steps/min, can effectively contribute to BP improvement in Chinese Han occupational populations with sedentary lifestyles. Moreover, we identified a significant dose‐effect relationship between changes in SBP and effective steps, but not in DBP.

The key focus of this trial was to motivate all participants to alter their sedentary lifestyles. But for most people, changing their routine habits may be difficult. However, since walking is an essential daily activity, the use of walking‐based aerobic exercises, such as brisk walking, is an ideal intervention to encourage adherence to moderate‐intensity movement suitable for most physically inactive people.^19^ Indeed, this study shows that a brisk walking intervention may be applicable to all occupational populations with sedentary lifestyles, as it has ideal adaptability to the physical performance and cardiovascular function of the participants. During the intervention period, participants complied well with exercise requirements, and exhibited high exercise enthusiasm; accumulating a drop‐out rate of only 14.2%. Approximately 4000–12 000 effective steps/day were performed by 73.1% of participants, indicating that a majority were able to adhere to intervention recommendations; 26.9% of participants exceeded the recommended number of effective steps. The BP benefits from engaging in a volume of walking greater than recommended by the intervention require careful consideration.

This study is based on open‐ended walking interventions in a real world, so that their exercise is highly independent and random. In the statistics and analysis of the dose‐effect relationship, the effective steps that they actually walked daily were divided into four levels of 4000‐ < 8000, 8000‐ < 10 000, 10 000–12 000, and > 12 000 steps/day, and observed their effect on BP, as well as their differences. We found that for both male and female participants, an effective steps level of more than 12 000 steps per day appeared to be detrimental in the control of SBP as an increase in the mean SBP level was demonstrated in these participants. In order to clarify whether excessive exercise is beneficial to control BP, the effective steps > 12 000 steps/day, which was beyond physical activity recommendations, was considered as the reference group. We found that 4000‐ < 8000, 8000‐ < 10 000, and 10000–12 000 steps/day had significantly better effect on reducing SBP than that by > 12 000 steps/day for all participants. In the male cohort, only the effective steps range from 4000 to < 8000 steps/day showed a better reduction in SBP; while in the female cohort, the effect of reducing SBP at 8000 to < 10 000 steps/day was better. Therefore, regardless of sex, the effective steps level of > 12 000 steps per day did not show a better reduction of SBP. These results further suggest that more than 12 000 effective steps per day did not show better control of SBP. However, the increase in SBP caused by exercise is one of the major contribution of cardiovascular and cerebrovascular events.^20^ Therefore, it is wise to avoid excessive exercise, especially if it is too strenuous. Additionally, the DBP of women decreased significantly only between 4000 and < 10 000 steps per day. This sex‐based difference in DBP may be related to the baseline DBP level; the baseline DBP in men seemed to be higher than that in women. It may also be that different sexes have different responses to the amount of exercise; however, more research is needed regarding this.

Brisk walking significantly reduced BP in hypertensive patients. Additionally, we further divided the participants into hypertensive and non‐hypertensive cohorts for discussion. For participants with hypertension at baseline, different exercise levels demonstrated a significant decrease in SBP and DBP, with an average decrease of 5.7 to 11.8 mm Hg and 5.1 to 10.9 mm Hg, respectively. The results further confirm that exercise is beneficial for controlling BP in patients with hypertension, and the effect of reducing BP is similar to that of recent meta‐analyses,^21^, ^22^ that is, aerobic exercise intervention can reduce SBP by 4.7 to 9.36 mm Hg and DBP by 3.2 to 5.93 mm Hg in the Asian hypertensive population. Meanwhile, the multiple linear regression model was used to analyze the relationship between BP changes and exercise in 117 patients with hypertension, and the confounding factors were adjusted. It showed that the effective steps that ranged from 4000 to < 8000 steps/day was better than > 12 000 steps/day in reducing SBP. This suggests that for patients with hypertension, brisk walking (100–150 steps/min) of 4000 to 8000 steps/day has the ideal effect on reducing SBP, while more exercise has a negligible effect on reducing SBP. For the non‐hypertensive cohort, the main outcome of exercise is to maintain the BP level. The effective steps ranged from 8000 to < 10 000 steps/day, and SBP was significantly decreased (an average decrease of 2.2 mm Hg); while more than 12 000 steps/day, SBP was significantly increased (an average increase of 2.0 mm Hg). The results of exercise intervention in non‐hypertensive people also suggest that excessive exercise (effective steps over 12 000 steps per day) is not beneficial for BP management in normotensive patients. The results of the multiple linear regression model for the non‐hypertensive cohort further showed that brisk walking of 4000 to 12 000 steps/day appeared to be more beneficial for BP control than excessive exercise. Thus, walking more than the recommended amount offered no additional BP control benefits. This result was similar to that reported in a systematic review by Cornelissen and coworkers,^23^ in which 30–45 min of moderate‐intensity aerobic exercise per day (equivalent to 3000–7000 steps/day of brisk walking) was reported to better control BP than greater amounts of exercise.

There are several possible mechanisms of the lesser effects of greater exercise on lowering BP. Firstly, we have to mention the formation mechanism of systolic pressure, that is, the rapid injection of blood in the ventricles of the heart during contraction, resulting in lateral pressure on the blood vessel walls. In order to maintain BP during exercise, the heart has to pump out more blood (cardiac output). Greater exercise, either intense or prolonged, places greater stress on the cardiovascular system and increases cardiac output. This results in an adaptive change in the heart's structure known as ventricular hypertrophy or dilation. Such conditions have been reported in studies involving athlete.^24^, ^25^, ^26^ But whether the change is physiological or pathological remains to be seen. In addition, this may be because the maintenance of BP during exercise depends on the excitement of sympathetic nerves. Long‐term exercise keeps the sympathetic nerves at a high level of excitement for a long time, so that the excitability of sympathetic nerves at rest may also increase adaptively.^27^, ^28^, ^29^ The SBP of individuals who exercised a lot was not reduced much or increased instead. However, a clearer mechanism still needs to be further verified by more studies.

Interestingly, increasing the amount of exercise did not lead to better SBP control, regardless of sex and presence of hypertension. Moreover, uncontrolled excessive exercise may increase the risk of exercise injuries or cardiovascular events. Additionally, excessive exercise may lead to less adherence to exercise routines, leading to exercise drop‐out. As mentioned in the review by Berge and coworkers,^30^ the incidence of hypertension in athletes with a large amount of exercise training is significantly higher compared to athletes with a small amount; therefore, greater amount of exercise is not necessarily better for BP control.

This study has practical implications for BP management in Chinese occupational populations with sedentary lifestyles, as it provides evidence for the appropriate amount of exercise required to control BP across sexes and different BP status groups. Several limitations exist in this study. First, participants did not exercise strictly in accordance with the amount recommended in the intervention, often blindly increasing their amount of walking; thus, resulting in a relatively small percentage of participants performing 4000–8000 steps/day. In addition, participants’ salt intake, smoking, and alcohol consumption were not required or recorded; and only a pre‐intervention health education on the risk factors of hypertension and related diet was conducted for all patients.

## 5 CONCLUSIONS

Exercise in the form of brisk walking may effectively improve BP control in Chinese Han occupational populations with sedentary lifestyles. A significant dose‐effect relationship was found between exercise and BP, with the highest exercise level seems to be detrimental to the control of SBP, especially in individuals with a history of hypertension.

## FUNDING

This study was Supported by the National Key R&D Program of China (2016YFD0400603) and the National Science and Technology Support Program (2013BAI06B05) from Ministry of Science and Technology of People‘s Repubilc of China. The funders had no role in study design, data collection and analysis, decision to publish, or preparation of the manuscript.

## CONFLICT OF INTEREST

The authors have declared that no conflict of interest exists.

## AUTHOR CONTRIBUTIONS

Cuiqing CHANG MD, PhD, conceptualized the intervention design and contributed to funding acquisition, and contributed to the development of the trial methodology and guided the implementation. Yingxiang YU MS, Chengcheng GUO MD and Lan XIE BS performed the data collection. Yingxiang YU and Yifan WU MS carried out the statistical analyses. Yingxiang YU interpreted the data and drafted manuscript which was reviewed by Cuiqing CHANG. All authors read and approved the final manuscript.
